# Interferon gene therapy with nadofaragene firadenovec for bladder cancer: from bench to approval

**DOI:** 10.3389/fimmu.2023.1260498

**Published:** 2023-08-29

**Authors:** Alberto Martini, Côme Tholomier, Sharada Mokkapati, Colin P. N. Dinney

**Affiliations:** Department of Urology, The University of Texas MD Anderson Cancer Center, Houston, TX, United States

**Keywords:** bladder cancer, gene therapy, immunogene therapy, BCG unresponsive bladder cancer, novel medications

## Abstract

Bladder cancer is a prevalent malignancy with limited therapeutic options, particularly for patients who are unresponsive to Bacillus Calmette-Guérin (BCG). The approval of interferon-α (IFNα) gene therapy with nadofaragene firadenovec (Adstiladrin^®^), the first gene therapy for genitourinary malignancies, has provided a promising alternative. This article reviews the research and milestones that led to the development and approval of nadofaragene firadenovec. Bladder cancer is well-suited for gene therapy due to direct access to the bladder and the availability of urine and tissue samples for monitoring. Early challenges included effective gene transfer across the urothelium, which was overcome initially by modulating the expression of coxsackie/adenovirus receptor (CAR) and, ultimately, by disrupting the urothelial barrier with Syn3. Nadofaragene firadenovec is a modified adenoviral vector carrying the IFNα gene. Clinical trials have shown promising results, with high response rates and manageable adverse events. Ongoing research focuses on improving patient selection, identifying biomarkers for response prediction, exploring alternative vectors for enhanced transfection efficiency, and developing combination strategies targeting resistance mechanisms. The approval of nadofaragene firadenovec marks a significant milestone in the field of gene therapy for bladder cancer, and future developments hold promise for further enhancing its efficacy and impact.

## Introduction

Bladder cancer (BLCA) is the seventh most common male malignancy, and the tenth worldwide overall (1). Bladder cancer is classified according to the depth of invasion as either non-muscle invasive (NMIBC) or muscle invasive (MIBC) (2). The former accounts for approximately 70% of newly diagnosed bladder cancers. The current standard of care for treatment of NMIBC is transurethral resection, followed by intravesical therapy. Bacillus Calmette-Guérin (BCG) is the most effective intravesical therapy and is currently indicated for high-risk patients (3). Unfortunately, over time most patients recur with BCG-unresponsive disease which is an inherently resistant disease with limited therapeutic options beyond radical cystectomy. However, many patients refuse or are not optimal candidates for this procedure. Prior to the approval of nadofaragene firadenovec (Adstiladrin^®^), there were only two United States Food and Drug Administration (FDA) approved drugs for BCG-unresponsive NMIBC; valrubicin and pembrolizumab (3); neither of which have been widely embraced by the urology community, and alternatives to cystectomy remained a pressing unmet need. The release of the FDA guidelines for trial design for BCG-unresponsive NMIBC in 2018 which supported single-arm registration trials for this indication unleashed the clinical development for this indication with a plethora of alternatives to cystectomy, including various chemotherapeutic agents, toxins, and immunotherapy (4). Nadofaragene firadenovec was one of the first of these to receive FDA approval in December 2022 and is the first gene therapy approved for genitourinary malignancies (5).

In this review, we will outline the research and milestones from bench to bedside that ultimately led to the approval of nadofaragene firadenovec.

## Gene therapy: definitions and early development

Gene therapy is the introduction of nucleic acid into the hosts cells to achieve a therapeutic effect. According to the FDA’s definition, gene therapy includes products that function by transcribing and/or translating transferred genetic material or by integrating it into the host genome (6). These therapies are administered as nucleic acids, viruses, or genetically engineered microorganisms. Gene therapy can be targeted directly at tumor cells to restore mutated tumor suppressor functions or modulate the host’s immune response to generate an anti-tumor reaction, known as “immunogene therapy.” (7)

The literature describes three types of gene therapy: in-situ, *in-vivo*, and ex-vivo. *In-vivo* gene therapy involves systemic administration, usually through the bloodstream, using vectors specific to the targeted disease. It offers the advantage of being able to target cells that cannot be isolated from the body, such as brain cells. However, there are disadvantages, including non-specific contact between the vector and the host, as well as a higher risk of immunogenicity and inactivation by antibodies and active immunity. On the other hand, ex-vivo or *in-vitro* gene therapy involves removing target cells from the patient or donor, manipulating their genomes in the laboratory, and reintroducing them into the patient. This method is commonly used for blood diseases. Lastly, *in-situ* gene therapy involves administering the treatment directly into a specific organ or area of interest, such as tumor injection or through a pre-inserted catheter in organs like the heart or bladder or peritoneal and pleural cavities (6).

Early gene therapy studies focused on replacing a defective gene with a normal copy to treat genetically inherited diseases like cystic fibrosis or muscular dystrophy. However, current research primarily aims to utilize gene therapy for the treatment of human cancers. Commonly employed vectors in clinical trials include adenoviral, retroviral, herpes virus and naked plasmid vectors.

## Development of gene therapy for bladder cancer

Bladder cancer is ideally suited for gene therapy. The bladder is a cavity that provides direct contact between vector carrying the therapeutic gene and the tumor. We have relatively easy access to urine and tissue to monitor the effects of therapy and perform correlative studies that might link a biomarker expression with sensitivity or resistance. Furthermore, relevant animal models are available to optimize therapy. But despite these advantages for developing intravesical gene therapy the early trials were disappointing as effective gene transfer across the urothelium remained a challenge.

The susceptibility of cells to virally-mediated gene transfer depends on three key factors: the presence of specific viral receptors on the cell surface, the requirement for episomal or viral integration to express the desired gene, and the existence of physical barriers hindering viral attachment (8).

Human epithelial cells, including urothelial cell carcinoma cells, are particularly receptive to adenoviral infection due to the ubiquitous expression of the coxsackie/adenovirus receptor (CAR) (9, 10). Adenovirus interacts with CAR, leading to intracellular incorporation of the virus and subsequent expression of the transgene. BLCA cell lines lacking CAR exhibit resistance to adenoviral infection and gene therapy (11). To overcome this limitation, researchers explored modulating CAR expression. Studies demonstrated that CAR expression can be increased through the addition of sodium butyrate, and histone acetylation was found to upregulate CAR expression in bladder cancer cells (12). This opened up possibilities for pharmacological modulation using histone deacetylase inhibitors like valproic acid, which effectively increased CAR expression in the T24 bladder cancer cell line both *in vitro* and *in vivo (* 13).

Transfection of the urothelium was the most daunting challenge to intravesical gene therapy, independent of the presence of appropriate cellular receptors for the selected vector. The urothelium is protected by a glycocalyx, including the glycosaminoglycan (GAG) layer, which serves various functions, including the prevention of bladder infections and in the case of gene therapy, the prevention of bladder infection by viral vectors (8). One common approach is to use chemicals to disrupt the urothelial barrier to enhance viral uptake. Initial success was reported using ethanol and acetone. However, adverse effects including hemorrhagic cystitis and stone formation limited its utility. In pursuit of a safer and more clinically useful transduction-enhancing agent the detergent Big CHAP [N,N0 -bis(3-D-gluconamidopropyl)cholamide] was evaluated in intravesical gene therapy (14). Ironically, the commercially available Big CHAP’s yielded inconsistent results, and it was an impurity in one of the formularies that facilitated effective gene transfer. This impurity was the third identified, hence it was named Syn3. Syn3 is an anionic detergent which was identified as a contaminant in only certain lots of Big CAHP. Syn3 enhanced transgene expression in urothelium and resembled BigCHAP except for a substitution of a second cholyl group for one of the gluconic acid moieties. A more soluble form of Syn3 was synthesized at Canji by substituting disaccharide lactobionic acid for the gluconic acid to increase its solubility (14). This discovery of Syn3 laid the foundation for the development of adenoviral interferon gene therapy for BLCA (15, 16).

## Interferon gene therapy for bladder cancer with nadofaragene firadenovec

**Figure 1:**
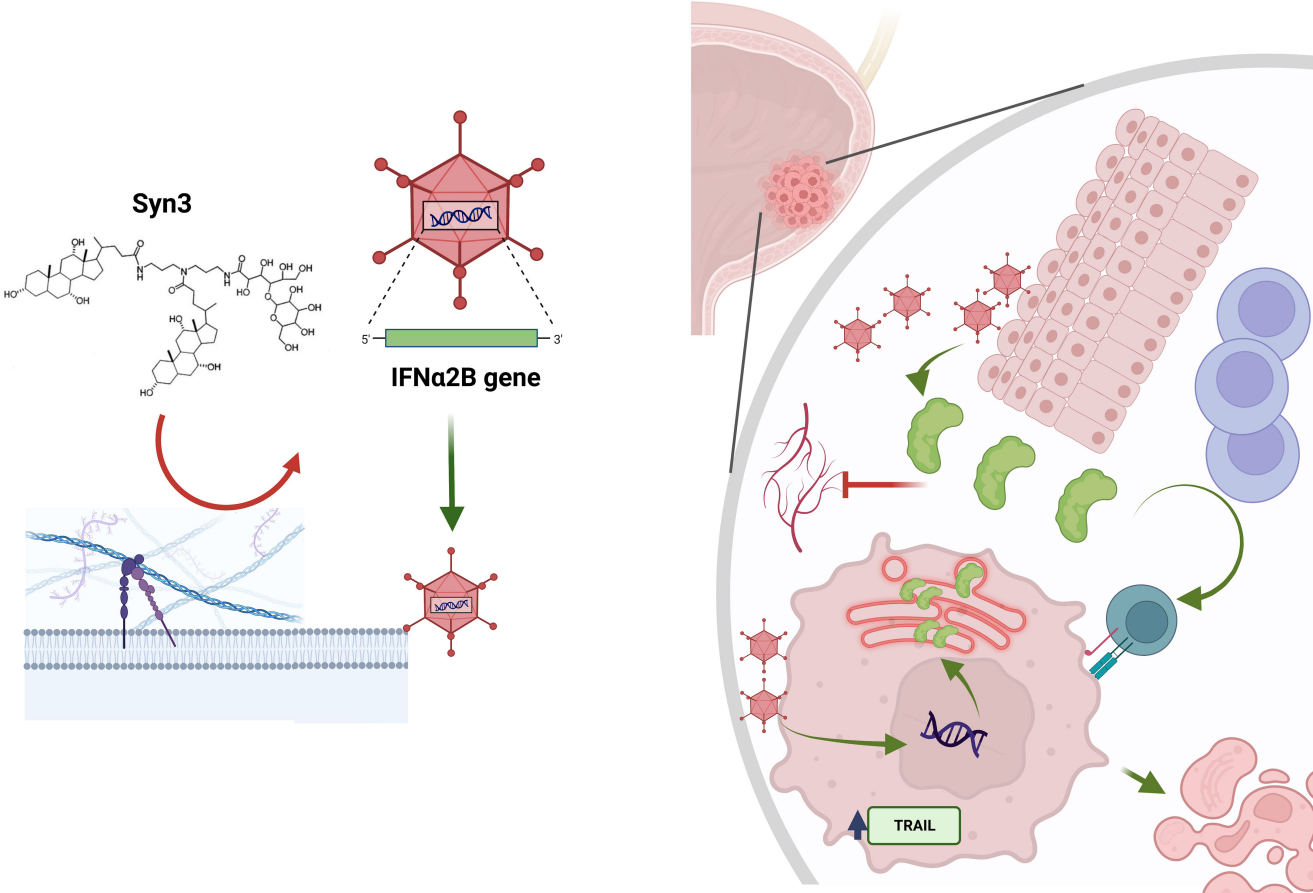
Mechanism of action of nadofaragene firadenovec and Syn3. Syn3 disrupts the bladder glycocalyx, allowing for the adenoviral vector to bind to its coxsackie-adenoviral receptor (not shown). The viral vector can transfect normal cells and cancerous cells. In the latter, interferon alpha (IFNα) synthesis can induce endoplasmic reticulum stress (in red within the tumor cell), which leads to apoptosis *via* caspase 4 activation, or to TNF-related apoptosis-inducing ligand (TRAIL) activation, which ultimately leads to apoptosis. The transfected normal cells produce IFNα. This phenomenon is known as the “bystander effect”. IFNα is released in the urine, where it can be measured, and in the extracellular matrix. Here, IFNα is responsible for T cell recruitment and activation and also decreased angiogenesis.

Interferon-α (IFNα) gene therapy was pursued due to the recognized pleiotropic anti-tumor activity of IFNα. IFNα has both antiproliferative activity and directly cytotoxic through TRAIL-induced cytotoxicity (17) and through the accumulation of interferon protein in the endoplasmic reticulum (ER) leading to caspase 4 activation and, ultimately, cell death (18). Notably, the phenomenon of ER stress was not observed in normal urothelial cells (18). IFNα also mediates an anti-angiogenic effect (19–21). Recently there has been renewed interest in interferon’s role in mediating an immune response. It is involved in antigen recognition and processing, leading to T-cell, natural killer and dendritic cell activation (17, 19–21). Clinical studies demonstrated that intravesical IFNα protein was ineffective largely due to insufficient exposure and transient IFNα availability after bladder instillation. rAd-IFNα/Syn3 was developed to overcome this limitation by producing high, sustained IFNα levels in the urine and bladder tissue (22, 23), (**Figure 1**).

Preclinical development of rAd-IFNα/Syn3 proceeded through a collaboration between Canji and investigators from MD Anderson Cancer Center. Together they showed that rAd-IFNα/Syn3 induced the regression of human bladder cancer growing in athymic nude mice (22–24). This was accompanied by sustained high urine and tissue IFNα levels (22–24). Re-dosing at 90 days was effective in restoring urine IFNα levels, and this schedule was adopted for clinical development. rAd-IFNα/Syn3 demonstrated both a direct and a bystander anti-tumor effect. The bystander effect is mediated by cancerous and normal urothelial cells, that, upon transfection, produce and release IFNα. No major toxicity in rodent or primate studies was noted and this compelling preclinical data supported the clinical development of this agent (22, 23).

## Clinical trials

The findings of the initial phase I clinical trial, which examined the safety and toxicity of intravesical rAd-IFNα/Syn3, were published in 2013 (25). In this study, patients with NMIBC who experienced recurrence after at least two cycles of BCG treatment were enrolled. The patients were treated with intravesical rAd-IFNα/Syn3 according to a standard dose-escalation design. Adverse events were monitored daily for up to five days after treatment, and at 12 weeks post-treatment patients underwent cystoscopy with bladder biopsy and urine cytology. A total of 17 patients participated in the study, and no dose-limiting toxicities or significant treatment-related adverse events (AEs) were observed (25). The most commonly reported AEs included urinary urgency, headache, fatigue, and nausea. Anticholinergic therapy effectively managed lower urinary tract symptoms that were experienced by 88% of the patients. The efficacy of gene transfer was demonstrated by measuring the levels of IFNα protein in the urine. All dosing cohorts, except for the lowest dose, demonstrated detectable levels of IFNα protein. Among the 14 patients who received effective doses and had confirmed gene transfer indicated by detectable urinary IFNα, six (43%) achieved a complete response (CR) at three months, with two remaining disease-free at 29 and 39 months (25). A Phase Ib study was designed to determine whether a second instillation 3 days after initial treatment produced higher and more durable urine IFN levels (26). The study indicated that a second instillation did not lead to improved IFNα production (26).

In the phase II study, conducted by the Society of Urologic Oncology Clinical Trials Consortium (SUO-CTC) between 2012 and 2015, 40 patients from 13 centers with high-grade BCG-refractory or relapsed NMIBC (Ta, Tis, T1) were randomly assigned to receive either a low dose or high dose of intravesical rAd-IFNα/Syn3 (27). Patients free from recurrence were dosed every three months for one year, followed by a study-mandated biopsy from the index tumor site and at least five random bladder biopsies from specific locations. The primary endpoint of the phase II trial was to achieve 25% freedom from high-grade recurrence at 12 months. Effective gene transfer was observed in all patients, as evidenced by measurable levels of IFNα-2b in their urine. No significant difference was found between the two dosing arms, although the median time to recurrence slightly favored the high-dose group (6.5 months vs. 3.5 months). Adverse events were manageable and the treatment was well tolerated. Importantly, rAdIFNα/Syn3 demonstrated efficacy for both HGTa/T1 and CIS± HGTa/T1. Subgroup analysis revealed a 50% high-grade recurrence-free survival (HG RFS) rate for HGTa/T1 tumors at 12 months and a 30% HG RFS rate for CIS. This is particularly significant because of the limited treatment options available for patients with CIS. At the time of the trial valrubicin was the only approved agent at the time for BCG-refractory CIS, with an approximate 10% 12-month RFS rate (27).

The phase III study, again performed by the SUO-CTC, enrolled 157 patients with BCG-unresponsive NMIBC across 33 centers in the US (28). The primary endpoint of the study was met as 53% of patients with CIS ± Ta/T1 BCG-unresponsive NMIBC achieved a CR at three months, and 24% maintained this at 12 months (28). Patients with HGTa/T1 tumors achieved a 73% high-grade recurrence-free (HGRF) survival rate at three months and a 44% HGRF survival rate at 12 months. It is notable that 10% of the patients who were clinically free from recurrence were found to have occult CIS on the 12-month end of study biopsy. The most commonly reported AEs were fatigue, bladder spasms, discharge around the catheter, urgency in urination, hematuria, chills, fever, headache, painful urination, urinary tract infection, and diarrhea. Only one grade 4 and no Grade 5 treatment-related AEs were reported in the study. Treatment-related AEs were transient, lasting less than two days on average, except for fatigue, which had a median duration of 11 days, and urinary frequency, which had a median duration of 41 days. The rate of discontinuation due to treatment-related AEs was 1.9% (28). Nanofaragene firadenovec was approved by the FDA in December 2022 on the basis of the SUO-CTCs Phase III trial.

## Future directions of interferon gene therapy

The efficacy and safety of nadofaragene firadenovec was confirmed by the Phase 3 trial, and while the development to date has been encouraging there is opportunity to enhance IFNα gene therapy. One approach is to improve patient selection by identifying patient characteristics or biomarkers that predict sensitivity or resistance. This would improve the response rate by selecting likely responders for treatment. Given the paucity of tissue available from patients with NMIBC the focus is on the development of urine and serum biomarkers. One promising biomarker identified in the Phase II and III trials was the induction of systemic anti-adenoviral antibodies which predicted for the durability of a favorable clinical response to nadofaragene firadenovec (29).Pre-treatment anti-adenoviral titers were not predictive of therapeutic response to Adstiladrin^®^ and did not limit the effectiveness of nadofaragene firadenovec. The induction of anti-adenoviral titers and the fold-change from baseline predicted for a durable response in patients who achieved a CR at 3 months. This biomarker is predictive rather than prognostic since absolute pre-treatment titers did not correlate with response (29). Other promising biomarkers under development include urinary cytokines, miRNA, and DNA alterations.

Another opportunity for development is to identify alternative vectors that improve transfection efficiency to provide more durable clinical responses. One of the possible limitations of rAdIFNα/Syn3 is the transient transgene expression of IFNα due to adenoviral immunogenicity. To overcome this potential limitation, lentiviral vectors (LV) were tested in preclinical models as they provide more stable transgene expression and are less immunogenic than adenoviruses. LV vectors expressing β-gal or IFNα have been shown to stably transduce murine bladder cancer cell lines and normal bladder urothelium (30). In preclinical studies, LV- IFNα led to specific immune modulation, inhibited angiogenesis, and demonstrated significant anti-tumor activity in multiple animal models.

Another opportunity for improving IFNα gene therapy is to develop novel combination strategies targeting resistance mechanisms. In the studies evaluating LV- IFNα gene therapy, several clinically actionable targets including PD-L1, and the EGFR were identified and warrant further study in combination with interferon gene therapy (30). Studies focusing on using single cell and spatial transcriptomics in multiple murine models are underway to identify additional resistance mechanisms and clinically actionable pathways.

## Conclusions

Nadofaragene firadenovec gene therapy for BCG-unresponsive NMIBC is the first gene therapy approved for a urological disease by the FDA. Its development from preclinical studies through the Phase III trial and final approval spanned over three decades. Responses were durable and moving forward it is critical to identify the appropriate candidates for interferon gene therapy, as well as approaches to enhance its impact. Recent efforts have focused on the identification of biomarkers that predict response and resistance and on strategies that combine interferon gene therapy with other therapeutics, either intravesical or systemic, including chemotherapy and immunotherapy.

## Author contributions

AM: Conceptualization, Writing – original draft. CT: Validation, Writing – review & editing. SM: Supervision, Validation, Writing – review & editing. CD: Conceptualization, Funding acquisition, Investigation, Project administration, Supervision, Validation, Writing – review & editing.
